# Pharmacokinetic and Metabolic Characteristics of Herb-Derived Khellactone Derivatives, A Class of Anti-HIV and Anti-Hypertensive: A Review

**DOI:** 10.3390/molecules21030314

**Published:** 2016-03-08

**Authors:** Wanghui Jing, Ruilin Liu, Wei Du, Zhimin Luo, Pengqi Guo, Ting Zhang, Aiguo Zeng, Chun Chang, Qiang Fu

**Affiliations:** School of Pharmacy, Health Science Center, Xi′an Jiaotong University, No. 76, Yanta West Street, Xi′an 710061, Shannxi, China; jingwanghui1987@163.com (W.J.); lrlxjtu1987@xjtu.edu.cn (R.L.); duwei82@xjtu.edu.cn (W.D.); luozm0930@163.com (Z.L.); guopengqi@stu.xjtu.edu.cn (P.G.); zhangting1234@stu.xjtu.edu.cn (T.Z.); agzeng@xjtu.edu.cn (A.Z.); changchun@xjtu.edu.cn (C.C.)

**Keywords:** khellactone derivatives, metabolism, pharmacokinetics, structure-metabolism relationship, drug-drug interactions, drug development

## Abstract

A vast number of structural modifications have been performed for khellactone derivatives (KDs) that have been widely concerned owing to their diverse biological properties, including anti-hypertension, anti-HIV, reversing P-glycoprotein (P-gp) mediated multidrug resistance, and anti-inflammation effects, to find the most active entity. However, extensive metabolism of KDs results in poor oral bioavailability, thus hindering the clinical trial performance of those components. The primary metabolic pathways have been revealed as hydrolysis, oxidation, acyl migration, and glucuronidation, while carboxylesterases and cytochrome P450 3A (CPY3A), as well as UDP-glucuronosyltransferases (UGTs) primarily mediate these metabolic pathways. Attention was mainly paid to the pharmacological features, therapeutic mechanisms and structure-activity relationships of KDs in previous reviews, whereas their pharmacokinetic and metabolic characteristics have seldom been discussed. In the present review, KDs’ metabolism and their pharmacokinetic properties are summarized. In addition, the structure-metabolism relationships of KDs and the potential drug-drug interactions (DDIs) induced by KDs were also extensively discussed. The polarity, the acyl groups substituted at C-3′ and C-4′ positions, the configuration of C-3′ and C-4′, and the moieties substituted at C-3 and C-4 positions play the determinant roles for the metabolic profiles of KDs. Contributions from CYP3A4, UGT1A1, P-gp, and multidrug resistance-associated protein 2 have been disclosed to be primary for the potential DDIs. The review is expected to provide meaningful information and helpful guidelines for the further development of KDs.

## 1. Introduction

A growing awareness of the determinant roles that pharmacokinetics and metabolism play for the therapeutic outcome of drugs *in vivo* has led many pharmaceutical companies to promote the assessment of pharmacokinetic and metabolic features as important objectives during new drug development [1]. In many cases, compounds that show promising activities *in vitro* are revealed later to be inactive *in vivo*, which may be attributed to their undesirable pharmacokinetic properties. Therefore, it is important to make in-depth understanding of: (1) the basic mechanisms participated in the absorption, distribution, metabolism and excretion (ADME) courses of drug-candidates; (2) the enzymes and transporters involved in the metabolism and transport of the drug-candidates; (3) the interactions between chemicals and the drug-metabolizing enzymes along with drug transporters, particularly, cytochrome P-450s (CYP450s) and P-glycoprotein (P-gp) which always offer the dominant contribution to the potential drug-drug interactions (DDIs); and (4) the activities and toxicities of the metabolites [1]. Moreover, corresponding to the structure-activity relationships (SARs), structure-metabolism relationships (SMRs) evaluation is becoming a feasible approach to supervise drug design based on calculated physicochemical parameters incorporating existing knowledge; hence, it has been widely concerned in drug development. 

**Figure 1:**
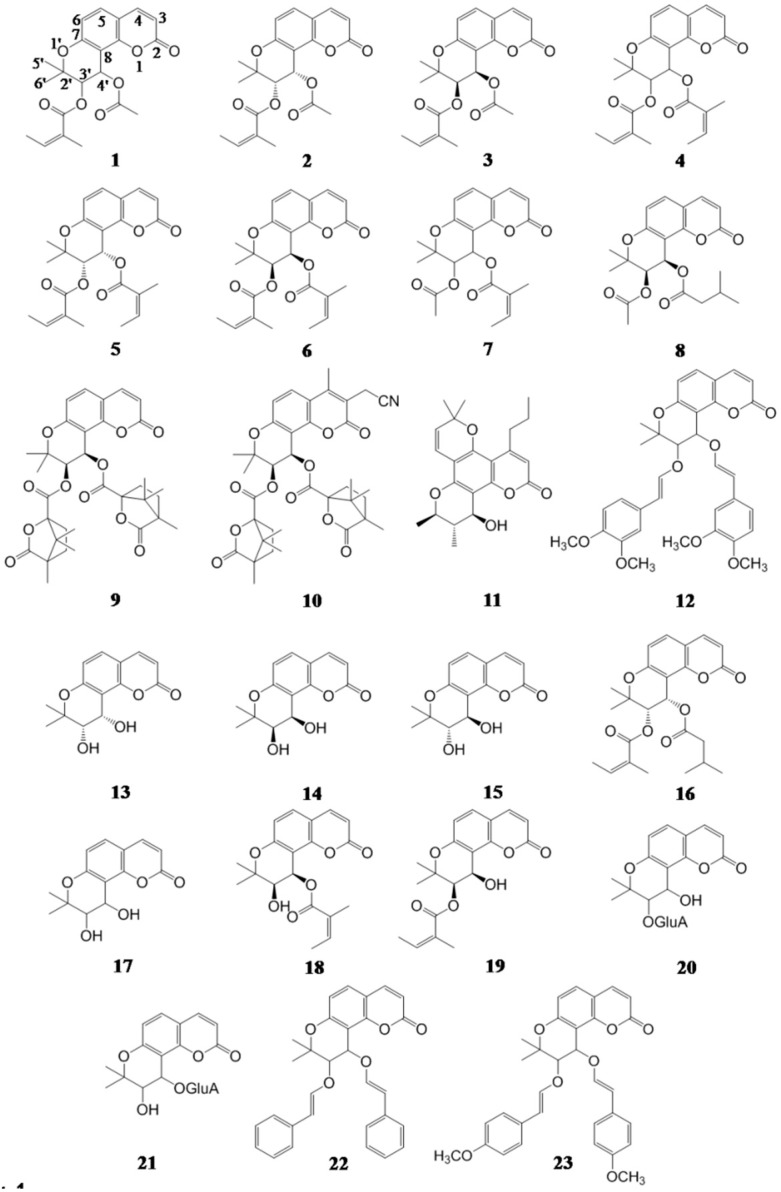
The chemical structures of khellactone derivatives **1**–**23** which exhibit promising activity.

So far, more than 50 natural khellactone derivatives (KDs) have been isolated and identified from plants, mainly from the genus *Peucedanum*, e.g., praeruptorin A (PA, **1**), (+)-praeruptorin A (*d*PA, **2**), (−)-praeruptorin A (*l*PA, **3**), praeruptorin B (PB, **4**), (+)-praeruptorin B (*d*PB, **5**), (−)-praeruptorin B (*l*PB, anomalin, **6**) and pteryxin (**7**) (Figure 1) [2]. Initially isolated from herbal medicines, a great number of structural modifications have been performed on KDs, aiming to develop novel agents for the treatment of Acquired Immune Deficiency Syndrome (AIDS) [3], hypertension [4] and P-gp-mediated multidrug resistance [5]. C-3′ and C-4′ of these KDs are usually observed as two stereogenic centers, suggesting that enantiomers and diastereoisomers could widely occur for KDs [6,7,8,9,10]. In view of the chiral preference of endogenous macromolecules, stereoselectivity should be a crucial issue for the pharmacokinetics and metabolism of KDs.

Suksdorfin (**8**) was isolated as an anti-HIV principle from *Lomatium suksdorfii* (S. Watson) J.M. Coult. & Rose (Umbelliferae) (“Beimei Qianhu”) [11]. Afterwards, an analog of suksdorfin, 3′,4′-*di*-*O*-(*S*)-camphanoyl-(+)-*cis*-khellactone (DCK, **9**) was demonstrated as a promising lead for anti-HIV agents by suppressing the production of double-stranded viral DNA from a single-stranded DNA intermediate, in stark contrast to current HIV-1 reverse transcriptase (RT) inhibitors that block the generation of single-stranded DNA from a RNA template [12,13]. This unique mechanism of action provides an opportunity to discover a novel non-nucleoside reverse transcriptase inhibitor (NNRTI) that remains effective against HIV-1 RT multi-drug resistant strains [14]. Because of the remarkable activities, systematic structural modifications of this famous leading compound have provided more than 150 KDs, including mono-, di-, and trisubstituted DCK-analogs [15], all of which maintain the (3′*R*,4′*R*)-configuration of suksdorfin, and their SARs have also been defined [16]. Recently, 3-cyano-methyl-4-methyl-DCK (CMDCK, **10**) showed the brightest prospects, because the introduction of cyanomethyl group can not only enhance the metabolic stability, but also offer a good H-bond acceptor and can favorably interact with Ser or Tyr aminoacid residues on the NNRTI binding site surface [17]. Moreover, it is worth noting that (+)-calanolide A (**11**), a compound that shares similar skeleton with KDs, is undergoing clinical trials as a candidate for the next generation of NNRTIs [18]. 

There are an increasing number of the pharmacological evaluations concerning the antihypertensive activity of *Peucedanum praeruptorum* Dunn. (Umbelliferae) (“Baihua Qianhu”) [19]. As the primary chemical homologue in this herbal medicine [6,7,20], KDs have been revealed to be responsible for this activity. Acting as the chemical indicator of *P. praeruptorum*, a combinatory mechanism has been revealed for the endothelium-dependent vasorelaxatory effect of PA enantiomers (*d*PA & *l*PA, **2** & **3**), mainly attributed to nitric oxide (NO) synthesis catalyzed by endothelial nitric oxide synthase (NOs) along with Ca^2+^ channel blocker, rather than K^+^ channel opener [21]. In order to obtain optimal structure responsible for this pharmacological feature, a vast number of KDs were synthesized by chemical modifying *d*PA, including *cis*- and *trans*-configurations to C-3′/C-4′. Bioactivity assays indicated that most KDs showed obvious Ca^2+^ antagonist activity, however, not comparable to *d*PA (**2**) [22,23]. Moreover, it was not surprised to discover that PA enantiomers (**2** and **3**) initiated different pharmacologic effects *in vitro* since the biological system has stereoselective preference which generally leads to chiral recognition during interactions between xenobiotics and endogenous macromolecules [24].

P-gp over-expression in tumor cells leads to multidrug resistance (MDR) and causes failure in cancer chemotherapy. The attempts to develop a new class of P-gp modulators have been made on KDs, aiming to reverse P-gp-MDR [5,25,26,27]. Mechanistic studies suggested that KDs could reverse P-gp-MDR through directly binding to substrate binding site(s) or allosteric site(s) on P-gp therefore impairing P-gp-mediated drug transport across cell membrane [25]. SARs evaluations suggest that MDR-reversal activity of KDs shows close correlation with the acyloxy substitution and the configurations of C-3′ and C-4′. Following a series of assessments, (±)-3′-*O*,4′-*O*-bis(3,4-dimethoxycinnamoyl)-*cis*-khellactone (**12**) bearing two methoxycynamoyloxy groups at the C-3′ and C-4′ along with (3′,4′)-*cis*-configuration, was revealed dramatic MDR-reversing activity *in vitro* and *in vivo*, indicating a promising prospect in medical usage [5,25]. 

In addition, some other pharmacological features have also been reported for KDs, such as anti-platelet aggregation [28,29], anti-inflammation [30,31,32], inducing differentiation and apoptosis of cancer cell [33,34], and cytotoxicity [35]. 

Above all, a great number of pharmacological evaluations have proved a bright prospect for KDs as drug-candidates. However, increasing number of articles revealed that their insufficient metabolic stability induced low oral bioavailability (F), thus limiting the further clinical development of KDs. The scopes of the current review include the summarization of the pharmacokinetic and metabolic properties of KDs, as well as the discussions concerning the structure-metabolism relationships of KDs and KDs-induced potential DDIs, aiming to provide meaningful information and helpful guidelines for the further development of KDs.

## 2. Pharmacokinetic Properties of Khellactone Derivatives

Many failures of drug candidates in development processes are the result of their undesirable pharmacokinetic properties, for instance poor absorption, undesirable half-lives (*t*_1/2_), and extensive first-pass effect. Regarding KDs, high lipophilicity (logP values calculated with ChemBioOffice 2008 are usually between 1 and 5) results in the good absorption, however, it leads to a low metabolic stability. Thus, it isn′t surprising to note that most KDs suffer from the low bioavailability due to the presence of various acyloxy groups at C-3′ and C-4′.

Till now, some investigations have been devoted to the preclinical pharmacokinetic profiles of KDs, and the pharmacokinetic parameters summarized in the literature [8,17,36,37,38,39,40,41] are collected in Table 1. In principle, the data in the table indicate that: (1) low bioavailability was observed for all KDs (lower than 26% for all investigated compounds), attributed to the low metabolic stability; (2) except for the end-hydrolyzed products, khellactones, the mean residence time (MRT_0–t_) values of KDs were less than 7 h; (3) except for the end-hydrolyzed products, their *t*_1/2_ values were less than 200 min when single compounds were administered, and the peak time (*t*_max_) values were less than 2 h; and (4) the other parameters, including apparent volume of distribution (V_d_), area under concentration-time curve (AUC), and peak concentration (*C*_max_), exhibited big variations due to the variegated structures. The plasma pharmacokinetic profile of PA (**1**) was characterized as a linear manner instead of non-linear profile, when this component was intravenously dosed (i.v.) [39]. At the meanwhile, after oral administration (i.g.) of either PA enantiomer or racemic PA (**1**), it is quite difficult to detect the prototypes, whereas *cis*-khellactone enantiomers (*d*CK and *l*CK, **13** & **14**) were observed as the dominant PA-derived components [38]. Similarly, *d*CK and *l*CK along with their diastereoisomer, (+)-*trans*-khellactone (*d*TK, **15**), were detected as the primary Peucedani Radix (the roots of *P. praeruptorum*) extract-derived components in rats following oral administration of the crude extract [8,42]. Even more, *l*PA (**3**) prototype disappeared rapidly following i.v. dosing (undetectable after 5 min) owing to the extensive hydrolysis catalyzed by the carboxylesterase(s) in rat plasma.

### 2.1. Absorption

Absorbability is one of the most important criteria for choosing new drug candidates for development. The drug absorption can be governed by a variety of biological and physicochemical factors, among which the two most important ones that determine both the extent and the rate of absorption are polarity and solubility. As elucidated in Table 1, KDs manifest quick absorption into the circulation system following oral administration.

Several *in vitro* platforms, e.g*.*, Caco-2 cell (human colon adenocarcinoma cell line) and Madin-Darby canine kidney epithelial cells transfected with the human MDR1 gene (MDCK-MDR1), have been well developed for permeability and absorption screening, of which Caco-2 cell monolayer model has been widely adopted as a preferable tool [43]. Yee [43] suggested that the overall ranking of compounds with the apparent permeability coefficient (*P*_app_) lower than 1 × 10^−6^ cm/s, between 1 and 10 × 10^−6^ cm/s, and higher than 10 × 10^−6^ cm/s can be classified as poorly (0%–20%), moderately (20%–70%), and well (70%–100%) absorbed candidates, respectively, while the efflux ratio (*P*_app BL→AP_ *vs.* *P*_app AP__→BL_) was adopted as the criterion to access the directional preference and to determine the transporter-mediated mechanism with a threshold of 2. The transports of PA (**1**), *d*PB (**5**), anomalin (*l*PB, **6**), and CMDCK (**10**) have been evaluated on Caco-2 cell monolayers model [44,45,46], among which the transport properties of PA were characterized under the help of a chiral HPLC-UV method and the parameters for both PA enantiomers (*d*PA & *l*PA, **2** and **3**) were obtained. The parameters are summarized in Table 2. Overall, all efflux ratios are less than 1.1; hence, the involvement of transport can be excluded. Apart from *d*PB (**5**), the other KDs exhibited good permeability (*P*_app_ great than 10 × 10^−6^) across the Caco-2 monolayers, agreeing well with the results from pharmacokinetic profiling (Table 1).

The carboxylesterase(s)-mediated enantiospecific hydrolysis is regarded to be responsible for the low recovery of *l*PA (**3**) in Caco-2 system, and subsequently resulted in enantioselective properties between *d*PA and *l*PA (**2** and **3**). In addition, it is interesting to find that an effective method was developed for the assessment of intestinal permeability of *d*PA (**2**), *l*PA (**3**), *d*PB (**5**), and *l*PB (**6**) on Caco-2 monolayer model using online guard column extraction coupled with tandem mass spectrometry [52].

### 2.2. Distribution

Drugs are often administered at a location distant from their intended site of action. Hence, to be effective, the drug must be absorbed and transported from the dosing site across several bio-membranes to reach the target tissue and the action domain. Penetrating cell membranes is a complicated course, which highly relies on the nature of the membrane and the physicochemical properties of the drug, such as ionization characters, hydrophobicity, number of hydrogen bonds, and molecular size [53].

Zhang *et al.* [39] investigated the distribution of PA (**1**) in a variety of biological samples, including plasma, urine, bile, tissues, and feces by developing a fast and sensitive LC-MS/MS method. The results demonstrated that PA is principally distributed in blood-supply tissues, such as heart, spleen, and lung with AUCs (areas under the curve) of 189%, 205%, and 134% of that in plasma after i.v. administration of PA (**1**), respectively, indicating that cardiovascular and respiratory systems are the main targets of PA (**1**). In addition, noticeable distribution of PA (**1**) in brain was confirmed because low polarity of PA permits it to cross the blood-brain barrier (BBB). In a comparison with kidney, faster elimination was observed for PA (**1**) in liver, which can be attributed to the extensive hepatic metabolism in the enzyme-enriched tissue. No long-term accumulation was observed for PA (**1**) in all tissues. Linear dynamics was manifested for PA (**1**) in all tissues following i.v. administration in dose range of 5–20 mg/kg. However, when PA (**1**) was orally administered, the metabolites rather than its parent compound were detected *in vivo*, resulting in that the oral distribution of PA (**1**) could not be characterized. At the meanwhile, the concentration of pteryxin (**7**), a regio-isomer of PA, in various tissues of mouse following the order: C_liver_ > C_brain_ > C_heart_ > C_kidney_, C_stomach_ > C_spleen_ > C_intestines_, and no long-term accumulation was observed for pteryxin in all tissues after oral treatment, coinciding with the findings obtained for PA. As expected, pteryxin bears great hydrophobicity (logP, 2.72, calculated with ChemBioOffice 2008), thus resulting in the extensive brain distribution via crossing BBB. In addition, Liang *et al.* [36] profiled the tissue distribution of *d*PB (**5**) in rats after intravenously treated, and claimed that the highest concentration was observed in the lung, followed by heart, liver, and kidney tissues, successively. *d*PB prototype (**5**) also can be detected in the brain, indicating that *d*PB (**5**, logP, 4.12, calculated with ChemBioOffice 2008) could overcome BBB after i.v. administration.

### 2.3. Excretion

Xenobiotics are generally eliminated from the body by metabolism and/or excretion. Both the liver and kidney offer key contributions for the excretion of drug prototypes and their metabolites. In principle, as aforementioned, metabolism was the primary pathway for the elimination of KDs; thus, it is reasonable to speculate that the recovery of prototypes in either feces or urine should be quite low.

The fecal and urinary samples after oral dosement of PA (**1**) were analyzed using LC-MS/MS in our group, the original form of PA (**1**) can be excreted through both pathways [54]. Whereas, low total recoveries were revealed for PA (**1**) within 24 h (0.120% in urine and 0.009% in feces), suggesting that quite a small portion of PA could be excreted in the original form through urine and feces, which might be caused by significant liver-mediated first pass effect [39]. In addition, a number of KDs′ prototypes were detected in the feces and urine following orally administered Peucedani Radix extract at a dose of 1000 mg/kg [42].

## 3. Metabolism of Khellactone Derivatives

Because the entire blood supply of the upper gastrointestinal tract passes through the liver prior to its arrival of the systemic circulation, the drug may be bio-transferred by the enzymes in either liver or intestine during the first passage of drug absorption. In general, the higher drug permeability and the greater metabolic clearance correspond to a higher lipophilicity, and thereby more extensive first-pass elimination [55]. KDs usually bear the khellactone skeleton with two acyloxy groups, one at the C-3′ position while the other is at C-4′, suggesting KDs always show high lipophilicity (logP usually among 1–5). Consequently, it is not surprising to note that KDs feature low metabolic stability and low bioavailablity in most cases. Not only the enzymes in liver and gut, but also the carboxylesterases in rat plasma were reported to possess the catalytic abilities for KDs [7,9,10,40,50,54,56,57,58]; however, the gut bacteria-catalyzed metabolism hasn′t been reported for this kind of coumarins [54]. Taking *l*PA (**3**) for instance, its prototype is undetectable even following intravenous administration, let alone oral treatment, while most portion of *d*PA (**2**) can be quickly metabolized into *l*CK (**14**) and some other metabolites owing to the extensive distribution of isozymes in both intestine and liver tissues.

Enzymatic kinetics of KDs has also been widely addressed, and the primary kinetic parameters documented in the literature are elucidated in Table 3. Corresponding to their concentration (remaining percentage)-time curves, most *t*_1/2_ values are lower than 30 min, indicating quick metabolism in human liver microsomes, human intestinal microsomes, rat liver microsomes, and recombination enzymes. The high levels of the *in vivo* intrinsic clearance (CL_int_), all of which were greater than 0.20 mL/min/mg, consolidate the observation of low bioavailability for KDs in rats. Michaelis constant (K_m_) values of PA (**1**), *d*PA (**2**), and CMDCK (**10**), were lower than 65 μmol/L, indicating relative high affinity with human intestinal microsomes, human liver microsomes, human CYP3A4, and rat liver microsomes, whereas V_max_ levels were higher than 0.25 pmol/min/mg.

**Figure 2:**
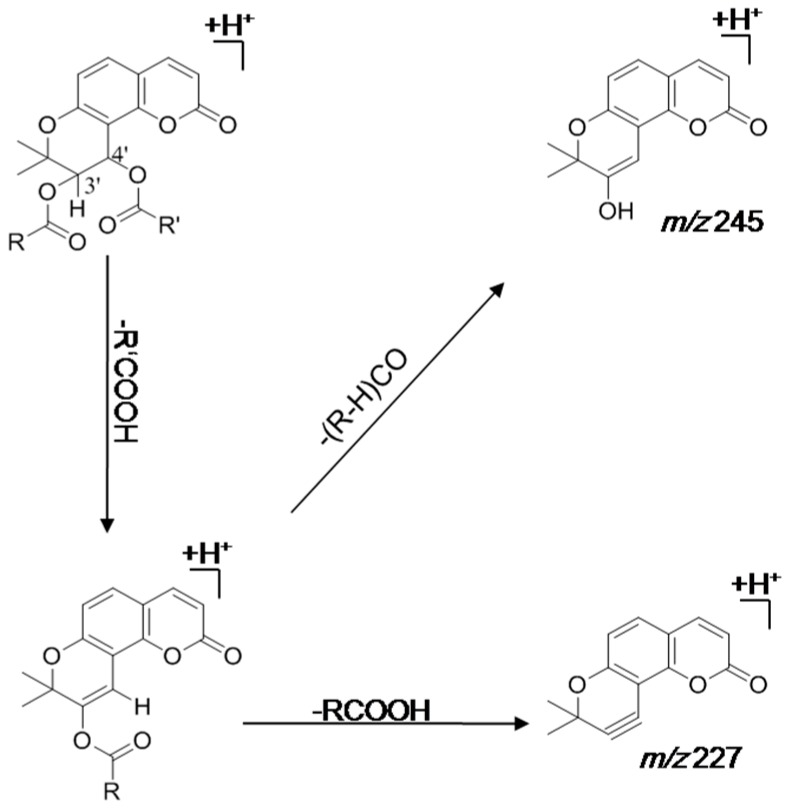
Proposed cracking rules of khellactone derivatives using electrospray ionization-tandem mass spectrometry.

Tandem mass spectrometric platforms, including ion trap, hybrid triple quadrupole-linear ion trap and time-of-flight mass spectrometry have been demonstrated as the reliable tools to plausibly identify the metabolites *in vitro* and *in vivo*. The fragmentation patterns of KDs have been previously proposed by various mass spectroscopic techniques [6,58]: initially, neutral loss takes place at the C-4′ position to afford a stable intermediate residue; the intermediate ion will subsequently cleave another neutral molecule from the C-3′ position to produce a diagnostic fragment ion at *m*/*z* 227 or remove an acyl group to yield the other characteristic signal at *m*/*z* 245. The cracking rules proposed for KDs are illustrated in Figure 2.

### 3.1. Metabolic Pathways of Khellactone Derivatives

#### 3.1.1. Hydrolysis of Khellactone Derivatives

**Figure 3:**
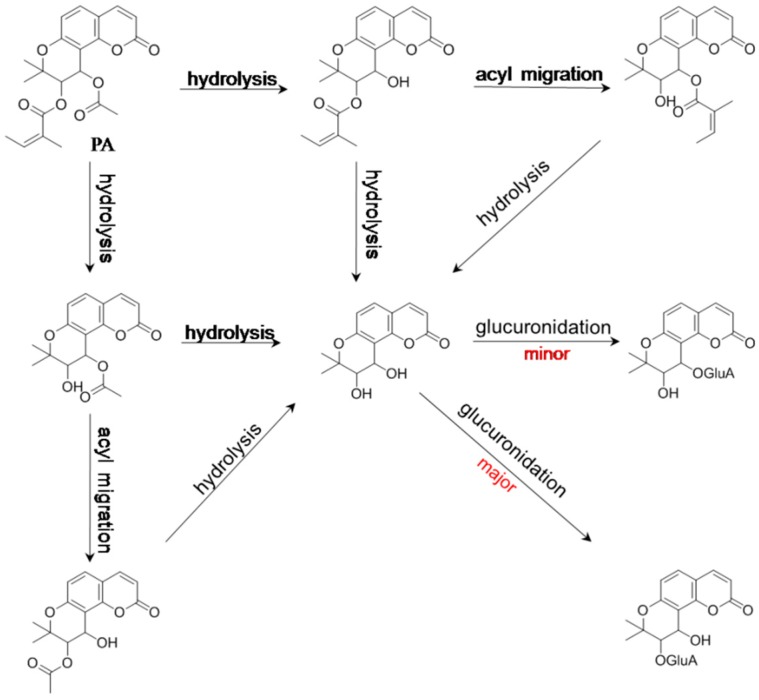
Hydrolysis-initiated metabolic pathways of praeruptorin A (PA), which is a natural khellactone derivative in Peucedani Radix.

In sight of the presences of two acyloxy groups, it is reasonable to believe that hydrolysis is a principal metabolic pathway for KDs (Figure 3).

On the other hand, phase II conjugation cannot occur owing to the absence of hydroxy/amino group in the chemical structures of most KDs. When the mass spectral profile of *d*PE (**16**) in liver microsomes was analyzed, a pair of hydrolyzed products was detected with similar mass spectrometric behaviors and the same molecular weight as 346 Da, and consequently, we made a preliminary speculation that one hydrolyzed metabolite was directly generated by hydrolysis, yet epimerization gave a birth to the other one [61]. However, this hypothesis was contrary to the observation of the sole end-hydrolyzed product, *cis*-khellactone (**17**). Aiming to validate our assumption for the generation of the isomers, an in-depth study was carried out in our group, thereafter. Two hydrolyzed metabolites of *l*PA (**3**), which shared identical molecular weight (344 Da), were isolated following incubation of *l*PA in fresh rat plasma, and then, the two hydrolyzed products were definitely identified as regio-isomers via a variety of spectroscopic and spectrometric techniques, suggesting intra-molecular acyl migration was responsible for the generation of the pair of regio-isomers (CAK-4 and CAK-3, **18** and **19**) [9], and also, for the observation of the paired hydrolyzed products of *d*PE (**16**). According to step-wise hydrolysis, the sole end-hydrolyzed product of PA (**1**) was afforded as *cis*-khellactone (**17**). Subsequently, further glucuronidation occurred for *cis*-khellactone (**17**) to afford a prominent *cis*-khellactone glucuronide (**17**) along with a minor one [54]. A tentative hypothesis was carried out in our previous report to address the definitely character of the *cis*-khellactone glucuronides based on the reactivity of the hydroxy groups at different sites [54]. It was well known that the nucleophilicity and stereo-conformation of hydroxyl groups play determinant roles for the glucuronidation preference [62]. The electron cloud density of the hydroxyl group at C-4′ of *cis*-khellactone could be down-regulated by the coumarin skeleton according to p,π-conjugation, suggesting that 3′-OH should show a higher reactivity for the glucuronidation than 4′-OH. Therefore, the major glucuronidated product could be plausibly identified as *cis*-khellactone-3′-glucuronide (**20**), whereas the minor one was thereby characterized as its regio-isomer, *cis*-khellactone-4′-glucuronide (**21**). Collectively, the metabolic pathways of PA (**1**) are summarized in Figure 3. On the other side, oxidative hydrolyzed product were detected for *d*PB (**5**) and *d*PE (**16**) [61]. 

**Figure 4:**
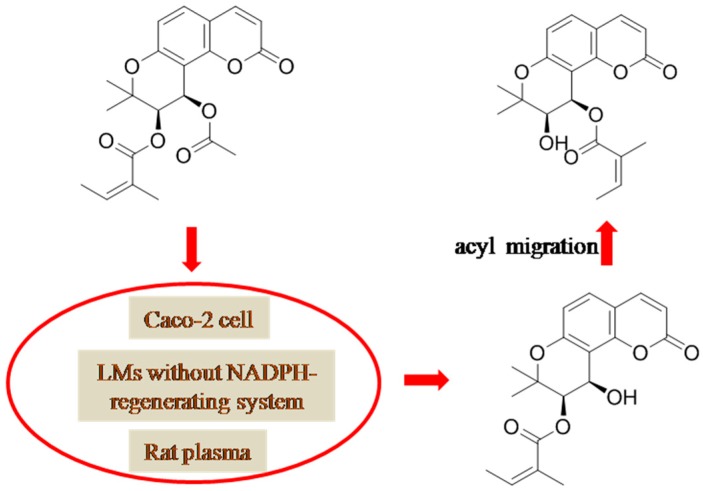
NADPH-independent hydrolysis and intra-molecular acyl migration of praeruptorin A (PA) in Caco-2 cells, in rat/human liver microsomes in the absence of NADPH-regenerating system, and in fresh rat plasma. LM: liver microsomes. The structures of the two *cis*-khellactone glucuronides were tentatively assigned on the basis of the speculation in [31].

NADPH-dependence is the criterion to judge the participation of CYP450s in the metabolism of xenobiotics. Although CYP450s catalyze most of the xenobiotic metabolism, the hydrolysis of *l*PA (**3**) was revealed to be partly mediated by the carboxylesterases in rat plasma, in Caco-2 cells, and in liver microsomes of rat/human in the absence of NADPH-regenerating system; however, *l*PA kept intact in human plasma (Figure 4). The contribution from human either carboxylesterases 1 or 2 (hCES1 or hCES2) was excluded using recombinant enzymes, suggesting that further study is called for to definitely identify the hydrolysis enzyme involved in the hydrolyzed cleavage of acyl group of *l*PA, which might also own the catalytic ability for the hydrolysis of some other KDs.

Species differences were observed for the hydrolysis of KDs due to the significant differences for the types and contents of enzymes between rats and human beings. It is necessary to mention that rat plasma carboxylesterases rather than human plasma carboxylesterases exhibit the catalytic ability for *l*PA (**3**) hydrolysis. The enzymes in human liver microsomes exhibited region preference for the angeloxy group at C-4′ of *d*PA, while hydrolysis could be initiated at either position in rat liver microsomes in the presence of NADPH-regenerating system [9]. Moreover, the generation rates of all hydrolyzed products in rat liver microsomes were different from those in human liver microsomes; in most case, the rat liver microsomes exhibit more powerful catalytic ability, in the other words, greater metabolic rates for KDs, than human liver microsomes.

#### 3.1.2. Oxidation of Khellactone Derivatives

The oxidation of coumarins has been extensively studied *in vivo* and *in vitro*, and coumarins can be transferred via a number of metabolic pathways, among which 3-hydroxylation and 3,4-epoxidation are demonstrated as the prominent pathways for coumarin in rat liver microsomes, and most coumarin oxidation are catalyzed predominately by CYP3A [63,64,65,66]. On the basis of this rule, Ruan *et al.* [57] suggested that PA underwent oxidation at the skeleton of coumarin. However, the oxidation site was finally placed on the side chains at C-3′ and C-4′ by our group and some other groups using the well proposed mass fragmentation patterns of KDs [9,54,58,59,61]. Step-wise oxidation was reported for KDs, from methyl group, to hydroxymethyl, then to aldehyde group, and even to carboxy group at last, such as the oxidation pathways of PA (**1**) [54] and CMDCK (**10**) [54,58].

In Ruan′s report [57], the mono-oxidation site was only speculated based on the neutral loss of an acetyl acid (CH_3_COOH, 60 Da) in the MS^2^ spectra of oxidized metabolites, while the product ion at *m*/*z* 227 corresponding to the loss of a C_4_H_6_OHCOONa (138 Da) molecule from the C-3′ position was overlooked, leading to an inappropriate judgment. Moreover, in order to unambiguously verify the oxidation site of *d*PA (**2**), scale-up incubation was performed for the incubation of *d*PA (**2**) in rat liver microsomes to yield abundant oxidized products. Following that, the incubated system, which contained the parent compound and metabolites, was entirely introduced for NMR measurement. The side chains were proved to be preferred for the regiospecific metabolism of *d*PA (**2**) after careful assignment of the NMR spectroscopic data. However, when cyanomethyl and methyl groups were introduced at C-3 and C-4, for instance CMDCK (**10**), oxidation could take place for cyanomethyl or methyl groups.

As expected, species discrepancies occurred not only for the elimination rate of the parent compounds, but also for the types and amounts of metabolites. Taking *d*PA as an example, only three oxidized products were detected in human liver microsomes; alternatively, six ones were detected in rat liver microsomes. Moreover, the content of each product exhibited significant variation between these two species. Collectively, the two species could generate obvious differences for both qualitative and quantitative aspects.

**Figure 5:**
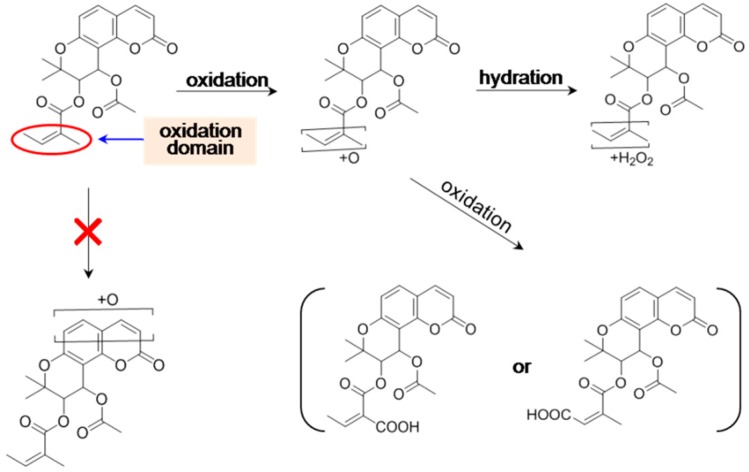
Proposed oxidation pathways of praeruptorin A *in vitro* and *in vivo*.

**Figure 6:**
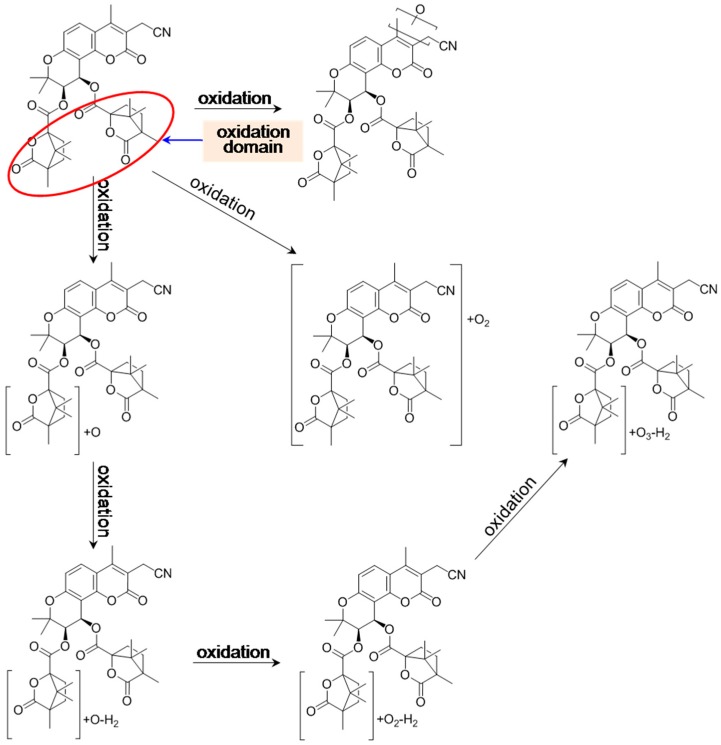
Proposed oxidation-initiated metabolic pathways of 3′,4′-*di*-*O*-(*S*)-camphanoyl-3-cyanomethyl-4-methyl-(+)-*cis*-khellactone (CMDCK) *in vitro*.

Above all, metabolic pathways, including stepwise hydrolysis, intramolecular acyl migration, glucuronidation, and stepwise oxidation were reported for KDs. The metabolic information is illustrated in Figure 3, Figure 4, Figure 5 and Figure 6.

### 3.2. Enzyme Involved in the Metabolism of KDs

The participation of CYP450s in the metabolism of KDs has been demonstrated by the NADPH-dependent manners for the generation of most metabolites. Zhuang *et al.* screened a panel of recombinant human CYP enzymes, including CYP1A2, CYP2B6, CYP2C8, CYP2C9, CYP2C19, CYP2D6, CYP3A4, and CYP3A5, as well as a series of chemical inhibitors, namely ketoconazole, troleandomycin, ritonavir, naphthoflavone, sulfaphenazole, tranlcypromine, and quinidine. Human CYP3A4 and CYP3A5 were proved to act the principal roles for the CMDCK (**10**) metabolism [58]. Identification of CYP450 isozymes involved in metabolism of *d*PA (**2**) was achieved in our groups by integrating chemical inhibitors and recombinant human CYP enzymes, as well as antibodies, and the findings consolidated the key role of human CYP3A4 for the KDs′ metabolism. Even more, Zhang *et al.* [37] clarified the contributions from rat CYP3A1 and CYP3A2 for the metabolism of PA (**1**) by comparing the pharmacokinetic profiles between normal rats and liver cirrhosis rats.

Carboxylesterases, enzymes that are widely distributed in the tissues and blood of mammals, mainly hydrolyze drugs containing ester and/or amide linkages, thus playing an important role in drug metabolism, especially for ester prodrugs. The contribution of hCES1 and hCES2 was excluded by screening recombinant enzymes for the hydrolysis of *l*PA (**3**). The hydrolysis of pteryxin in rat plasma was also reported in our previous article [42].

However, both carboxylesterases and UDP-glucuronosyltransferases (UGTs) that are involved in the hydrolysis and glucuronidation of KDs haven′t been definitely characterized, thus, further studies are required to address this issue. In addition, it should be taken into account that the rat plasma carboxylesterases are quite different from those of humans, hence, it is not reasonable to only use rats as the sole model animals to assess the preclinical pharmacokinetics of KDs. 

### 3.3. Structure-Metabolism Relationship

Structure-metabolism relationships (SMRs) evaluation is the one of the most important branches of structure-pharmacokinetics relationships investigation, owing that SMRs feature characterization could provide reliable information to obtain optimal structure of new drug with desirable metabolic features. Metabolic characterization has been performed for a great number of KDs. Herein, we aim to summarize both of the qualitative and quantitative information archived in the literature, and then to propose the SMRs for KDs.

As indicated above, KDs share a common core structure, and the differences just occur at the types of acyloxy groups substituted at C-3′ and C-4′ positions, except when structural modifications are performed at C-3 and C-4 of some KDs. Hence, we suggest that the substituents at C-3′ and C-4′ along with the configurations of C-3′ and C-4′ (*R* or *S*) play the key roles for SMRs. When moieties were introduced to C-3 and C-4, the groups could contribute to the hydrophobic coefficient (logP), and subsequently affect the metabolic stability.

Acyl migration was only observed for *cis*-khellactone derivatives that possess ester bonds at both C-3′ and C-4′ in the same configuration (*cis*-type) due to the space barrier in the case of *trans*-configuration. Until now, only *l*PA and pteryxin, which was identified as having a 3′*R*,4′*R*-configuration by our in-depth study (unpublished data), revealed carboxylesterase(*s*)-catalyzed hydrolysis; therefore, we speculate that carboxylesterase(s)-mediated hydrolysis occurs to KDs having a combination of angeloyl and acetyl substituents with *R*-configuration at C-3′ and C-4′. When hydrolysis has taken place, the skeleton structure would be generated as one of main hydrolyzed products and the absolute configuration of their respective parent compounds maintained. In the case of 3,4-unsubstituted KDs, oxidation only takes place at large (isovaleryl, angeloyl or camphanoyl) side chains, not at smaller (acetyl) substituents, e.g., PA enantiomers. More oxidized products were observed for *d*PA than *l*PA, tentatively suggesting that the (3*S*,4*S*)-configuration could enhance the oxidative ability. Otherwise, oxygen can be added onto the substituent(s) at C-3 and/or C-4 of 3,4-substituted KDs, for instance CMDCK (**10**) [58]. 

The metabolic stability of KDs exhibited a positive correlation with their hydrophilicity (logP values). When *d*PB and *d*PE were incubated with human liver microsomes or rat liver microsomes in parallel, the remaining percentage of *d*PE is less than that of *d*PB in either human liver microsomes or rat liver microsomes, corresponding to the higher hydrophilic level (logP value) for *d*PE. Meanwhile, 3′*R*,4′*R*-configuration was tentatively regarded exhibiting a better metabolic stability than its antipode based on the findings observed from *d*PA *vs.* *l*PA. On the other side, several series of mono- and disubstituted DCK derivatives were adopted to establish the quantitative SARs of DCK derivatives via assessment of the *in vitro* metabolic stabilities in human liver microsomes. The metabolism results indicated that all DCK derivatives underwent rapid oxidation on the lipophilic camphanoyl moieties and the two camphanoyl ester moieties were the determinants of the low metabolic stability, suggesting that structural alteration in these two ester moieties is a feasible way to improve the metabolic profiles of DCK derivatives. A cyano group showed good metabolic stability through improving the hydrophilicity of KDs when it was introduced onto C-3 site. Further SMRs evaluations are ongoing in our group to obtain some supervisory evidences for further structural modifications of KDs.

**Figure 7:**
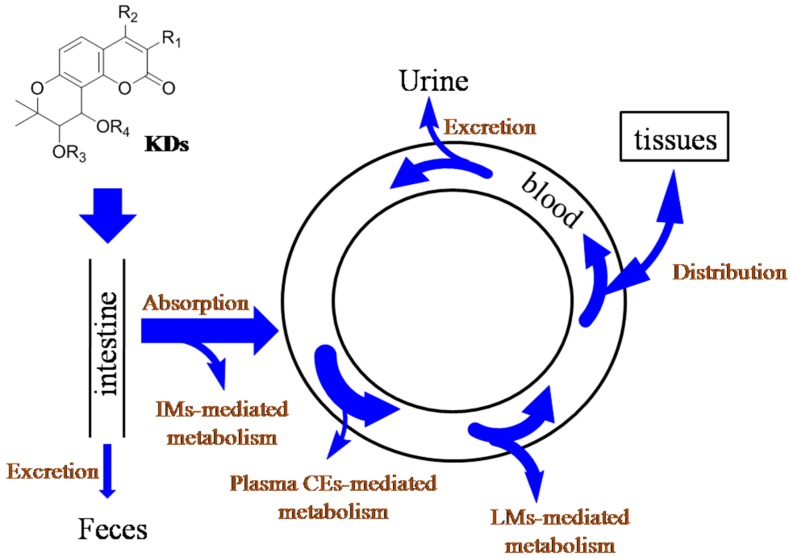
Schematic illustration of the absorption, metabolism, distribution and excretion (ADME) courses of khellactone derivatives following oral administration. IM: intestinal microsomes; LM: liver microsomes; plasma CEs: carboxylesterases.

Above all, the ADME courses of KDs following oral administration are summarized in Figure 7. Firstly, the intestinal barrier only slightly hinders the absorption of KDs, and intestinal bacteria could not mediate the metabolism of KDs. And then, the critical roles of intestinal microsomes, liver microsomes, and plasma carboxylesterases were demonstrated for the elimination of KDs from circulation system. At the meanwhile, urine-mediated excretion could be taken place for KDs. The wide tissue distribution could also impact the concentration of KDs in blood, in particular blood-supply tissues, such as heart, spleen, and lung, and KDs could cross BBB to achieve brain distribution. The attempts to improve the plasma concentration and oral bioavailability of KDs should pay attention to their crucial metabolism-mediated elimination.

## 4. Potential DDIs

Concomitant administration of several drugs is quite common and, indeed, is usually the situation in hospitalized patients. Whenever two or more drugs are administered over similar or overlapping time periods, DDIs might occur. Although DDIs can be explained by pharmacodynamic or pharmacokinetic effects, in many cases, the DDIs show a pharmacokinetic, rather than pharmacodynamic, basis. Interaction through mutual competitive inhibition among drugs is almost inevitable, owing that metabolism acts as a major route of drug elimination from the body, and also many drugs can compete for the same enzyme system, in particular CYP450s.

Inhibition and induction of CYP450 enzymes, such as CYP3A4, are probably the most common causes for DDIs. Several promising drug candidates have been withdrawn from the market attributing to the serious adverse effects as a result of CYP450s-mediated DDIs. Therefore, CYP450s-mediated DDIs have always been regarded as one of the major concerns for clinicians and patients. Besides metabolic interactions, it is also necessary to recognize that drugs that could regulate the protein expression of CYP450s may have a substantial contribution for drug interactions. 

Moreover, the involvement of transporters, in particular P-gp, for DDIs has been widely reported. Owing to its intracellular localization, the P-gp can limit cellular uptake of drugs from the blood circulation into the brain and placenta, and also from the gastrointestinal lumen into the enterocytes. P-gp can also enhance the elimination of drugs out of the hepatocytes, renal tubules, and intestinal epithelial cells into the adjacent luminal space. Therefore, there is a prominent role for P-gp to show a greater impact on drug transport. Like CYP450s, inhibition and induction of P-gp have been reported as the primary causes for DDIs.

Pregnane X receptor (PXR) and constitutive androstane receptor (CAR) are members of the orphan nuclear receptor subfamily, and they were originally defined as xenobiotic receptors, regulating the expression of drug-metabolizing enzymes and transporters as adaptive responses to prevent the accumulation of toxic chemicals in the body. During the last decade, mounting evidences suggested that PXR and CAR induce a broad spectrum of hepatic and intestinal genes involving in xenobiotic metabolism and transport. Those target genes include phase I enzymes CYPs (*i.e.*, CYP3A4, CYP2B6, CYP2Cs, and CYP2A6), phase II enzymes glutathione-transferases, UGTs, *i.e.*, UGT1A1, UGT1A6, and UGT1A9, and sulfotransferases (SULTs), as well as drug transporters, such as multidrug resistance protein 1 (MRP1), multidrug-resistance associated protein 2 (MRP2) and organic anion transporter polypeptide 2 (OATP2).

As xenobiotic receptors, another important feature of either PXR or CAR is the ability to recognize numerous chemical signals. A variety of structurally diverse CAR/PXR ligands have been reported, including pharmaceutical drugs, environmental pollutants, herbal medicines, dietary supplements, and endobiotics.

### 4.1. CYP450s and UGTs Mediated DDIs

Preliminary findings suggested that the total coumarin extract of Peucedani Radix, which is a KDs-enriched complexity, could down-regulate the activity of hepatic microsomal drug-metabolism enzymes in a dose-dependent manner, including CYP1A1, CYP2E, CYP2C11, and CYP2B1 in mice after oral administration, and subsequently regulate the metabolism of pentobarbital sodium [67]. Afterwards, Iwata *et al.* reported that the activity of CYP3A4 rather than CYP2D6 could be inhibited by the methanol extract of Peucedani Radix via co-incubation with probe substrates [68]; whereas, the inhibition could be relieved according to pre-incubation, attributing to the inactive metabolites generated from the extract by crucial metabolism [69]. In view of the prominent content of PA (**1**) in Peucedani Radix, it is tentatively suggested that PA could provide primary contribution for the CYP-activity inhibitive effect, whereas its metabolites couldn′t regulate the enzymatic activity. 

However, some contrary findings were obtained when single compounds were introduced to assess their influences on the CYP3A mRNA, protein expression, and functional activity. KDs, including PA (**1**), *d*PA (**2**), *d*PB (**5**), and *d*PE (**16**), could significantly enhance catalytic activity of CYP3A, particularly CYP3A4, through inducing the mRNA transcription along with protein expression [70,71,72,73]. As aforementioned, PXR and CAR are critical determinants of xenobiotics-induced CYP3A expression and they can generate crosstalk regulation on CYP3A transcription. PXR-/CAR-over expressed and untransfected human colon adenocarcinoma cells (LS174T) were assessed in parallel to clarify the roles of PXR and CAR for the increment of CYP3A4 activity. The results indicated that KDs can co-activate the CAR- and PXR-mediated pathways to co-regulate CYP3A expression. In-depth docking studies revealed that KDs can be readily docked into the ligand-binding cavity of PXR mainly through hydrogen bond formation and/or π–π interactions with the residues Ser247, Gln285, His407, and Arg401 [74].

Protein and mRNA expressions of UGT1A1 were determined by real-time PCR and western blotting assays after PA (**1**) and *d*PA (**2**) were incubated with HepG2 cells. In parallel, effects of PA (**1**) and *d*PA (**2**) on UGT1A1 mRNA and protein expressions were also measured after transient transfection of a specific CAR siRNA in HepG2 cells. Consequently, the UGT1A1 mRNA and protein expression levels could be significantly induced by either PA or *d*PA, whereas the mRNA and protein up-regulation of UGT1A1 could be attenuated by transient transfection of a specific CAR siRNA, suggesting the critical role of CAR for the inductions of UGT1A1 mRNA and protein expression.

Meanwhile, in the mRNA and protein expression regulation of several enzymes, competitive inhibition initiated by KDs was also reported for the drugs which are the substrates of CYP3A4. Preclinical assay was performed for the co-administration of ritonavir and CMDCK (**10**) to evaluate the potential competition between these two substrates of CYP3A4. Through inhibiting CYP3A enzymes in both of the intestine and liver tissues, the concomitant administration of ritonavir can significantly increase the bioavailability of CMDCK (**10**) and strengthen its plasma exposure, suggesting extensive CYP3A4-mediated competition between CMDCK (**10**) and ritonavir. Seldom have KDs been assessed for competitive inhibition, however, corresponding to the chemical structures of KDs, we propose that the acyl substitutes play the determinant roles for the competitive inhibition phenomena at the meanwhile of determining the metabolic types and rates.

### 4.2. Transporter-Mediated DDIs

The roles of transporter-mediated DDIs have been well defined, and protein expression regulation and activity inhibition were regarded as the two primary routes, of which activity inhibition can be mediated with competitive and non-competitive manners. During the absorption assessment of KDs using Caco-2 cell monolayers, the involvements of P-gp and the other prominent transporters were excluded, indicating that the competitive inhibition for transporters can be neglected. Alternatively, KDs could interfere in transporter action through non-competitive manner. Following an extensive herbal drug screening program, PA was revealed resensitizing potential for P-gp-mediated MDR (P-gp-MDR) cancer cells response to cancer drugs, thus being regarded as an inhibitor of P-gp. Subsequently, based on the promising performance, a series of structural modifications were carried out by Fong′s group [5,25,26,27,33,34] to yield a number of PA derivatives, one of which, (±)-3′-*O*,4′-*O*-dicinnamoyl-*cis*-khellactone (**22**), exhibited more potency than PA and verapamil (a definite inhibitor of P-gp) for the reversal of P-gp-mediated MDR. In P-gp-MDR cells, (±)-3′-*O*,4′-*O*-dicinnamoyl-*cis*-khellactone (**22**) could increase cellular accumulation of doxorubicin (a probe substrate for P-gp) without regulating the protein expression level of P-gp. In P-gp-enriched membrane fractions (±)-3′-*O*,4′-*O*-dicinnamoyl-*cis*-khellactone (**22**) could moderately stimulate basal P-gp-ATPase activity. However, this compound inhibited P-gp-ATPase activity stimulated by the standard substrates verapamil or progesterone via decreasing V_max_ value rather than K_m_ level. (±)-3′-*O*,4′-*O*-dicinnamoyl-*cis*-khellactone (**22**) could decrease reactivity of P-gp-specific antibody, agreeing well with the speculation of a non-competitive inhibition mode. Collectively, it was suggested that (±)-3′-*O*,4′-*O*-dicinnamoyl-*cis*-khellactone (**22**) binds simultaneously with substrates to P-gp but perhaps at an allosteric site, and thereby affects P-gp–substrate interactions. In general, two ATP-binding domains are also involved in the P-gp function of drug transport. Either the substrate binding sites and ATP-binding domains interact cooperatively as a functional unit; therefore, KDs could affect ATP hydrolysis, and subsequently suppress P-gp-mediated drug transport. Afterwards, a more superior inhibitor, (±)-3′-*O*,4′-*O*-bis(3,4-dimethoxy)-cinnamoyl-*cis*-khellactone (**12**), was screened out from numerous structural modification products. The coexistence of 3- and 4-methoxy groups at the cinnamoyl moieties remarkably enhanced the P-gp-inhibitory activity, whereas the lone existence of the 4-methoxy group on cinnamoyl reduced the activity. Contrary to (±)-3′-*O*,4′-*O*-dicinnamoyl-*cis*-khellactone (**22**), (±)-3′-*O*,4′-*O*-bis(3,4-dimethoxy)-cinnamoyl-*cis*-khellactone (**12**) promoted the binding of UIC2 antibody to P-gp to induce a conformational change of P-gp. In addition, although (±)-3′-*O*,4′-*O*-dicinnamoyl-*cis*-khellactone (**22**) could moderately stimulate the basal P-gp-ATPase activity, (±)-3′-*O*,4′-*O*-bis(3,4-dimethoxy)-cinnamoyl-*cis*-khellactone (**12**) significantly inhibited P-gp-ATPase activity. A pharmacophore search with verapamil-based template revealed that four functional groups of (±)-3′-*O*,4′-*O*-bis(3,4-dimethoxy)-cinnamoyl-*cis*-khellactone (**12**) could simultaneously participate in the interaction with P-gp whereas only three domains of (±)-3′-*O*,4′-*O*-dicinnamoyl-*cis*-khellactone (**22**) or (±)-3′-*O*,4′-*O*-bis(4-dimethoxy)-cinnamoyl-*cis*-khellactone (**23**) could be involved in the binding with P-gp. Above all, 3′-*O*,4′-*O*-aromatic acyl substituted KDs could serve as a new class of P-gp modulator through directly binding with substrate site(s) or allosteric site(s) on P-gp to hinder drug binding to P-gp, and consequently to slow down ATP hydrolysis and drug transport. The SARs′ characteristics of KDs were summarized from the Fong′s reports [5,25,26,27,33,34] as follows: (1) aromatic acyl groups contributed more than linear or branched aliphatic acyl group to MDR reversal activity of KDs; (2) *cis*-configured KDs exhibited higher MDR reversal potency than the *trans*-type KDs; and (3) 3,4-dimethoxyl substituted aromatic acyl groups, which might interact with P-gp as hydrogen bond accepter were more suitable than other groups for enhancing MDR reversing activity of KDs.

Multidrug resistance protein 2 (MRP2) belongs to the ATP-binding cassette (ABC) transporter family. It is one of the canalicular export pumps expressed on the apical membrane of polarized cells and can be extensively distributed in various tissues, including intestine, liver, and kidney. MRP2 plays an indispensable role in exporting a wide spectrum of organic anions, mainly conjugates of various toxins and carcinogens with glutathione (GSH), glucuronate, or sulfate. In order to assess the effect of KDs on MRP2, the changes in mRNA level, protein expression, and transport activity of MRP2 were determined by quantitative real-time PCR, western blotting, and the fluorescent MRP2-substrate 5-(6)-carboxy-2′,7′-dichlorofluorescein (CDF) uptake assay, respectively. In addition, the effects of CAR knockdown on MRP2mRNA and protein expression were also studied by transient transfection of a specific CAR siRNA. As a result, PA (**1**) and *d*PA (**2**) could significantly induce the MRP2mRNA and protein expression, and thereby enhanced the transport activity of MRP2. Moreover, mRNA and protein expression upregulations were attenuated by transient transfection of a specific CAR siRNA, suggesting that the upregulation of MRP2 was mediated by the CAR-pathway. Taken together, PA (**1**) and *d*PA (**2**) can significantly upregulate MRP2 expression via the CAR-mediated pathway *in vitro*.

X-ray crystallography demonstrated that PXR has a much larger ligand-binding pocket in comparison with other nuclear receptors (NRs), which enables PXR to bind a wide variety of ligands [75]. The ligand-dependent PXR activation has been shown to be species specific. For example, the antibiotic rifampicin is a potent PXR activator in humans and rabbits; whereas, it has little effect on the mouse or rat PXR. In contrary, the synthetic anti-glucocorticoid pregnenolone-16*α*-carbonitrile (PCN) can activate the mouse and rat PXR; however, it exhibits no effect on human PXR. In X-ray crystallography studies, CAR was shown to have a much smaller ligand-binding pocket than PXR [75]. Unique structural conformations were characterized that may explain the ligand-independent activities of CAR [76,77]. The ligand binding of CAR also shows species specificity. For example, 6-(4-chlorophenyl)imidazo[2,1-*b*][1,3]thiazole-5-carbaldehyde-*O*-(3,4-dichlorobenzyl)oxime (CITCO) is a potent agonist for the human CAR but not the mouse CAR, while 1,4-bis[2-(3,5-dichloropyridyloxy)]benzene (TCPOBOP) is more selective for mouse CAR than human CAR. The species specificity of CAR and PXR represents a challenge for suitable animal models to evaluate candidate human drugs.

In general, it is believed that endogenous CAR and PXR reside in the cytoplasm of hepatocytes [75,78]. Upon exposure to its agonist, for instance KDs, CAR, and PXR translocate from the cytoplasm to the nucleus of the cells. Then they bind to their DNA response elements as heterodimers with the retinoid X receptor (RXR). CAR could heterodimerize with RXR prior to binding with the promoter region of CYP3A4. The CAR/RXR complex binds to a sequence in the 5′-untranslated region of the gene that contains two copies of the nuclear receptor organized as ER6 and controls the expression of pre-mRNA. On the other hand, PXR is capable of dimerizing RXR to facilitate DNA binding specificity via two highly conserved zinc finger motifs as well as a P-Box motif and D-Box motif which allow the receptor to target and bind its xenobiotic response elements (XREs) located in the 5′ promoter region of PXR target genes.

The nuclear receptors, in particular CAR and PXR, are responsible for many important xenobiotic responses. Initially, KDs bind to the CAR and PXR in the cytoplasm, and then CAR and PXR translocate from the cytoplasm to the nucleus of the cells. After the formation of heterodimers or heterotetramers with RXR, the complexities will bind to their corresponding DNA sites to enhance the transcriptions of pre-mRNA, which, subsequently, can be translocated into cytoplasm, and subsequently to up-regulate the protein expression of CYP3A4, UGT1A1 and MRP2, but, not to affect the protein expression of p-gp.

**Figure 8:**
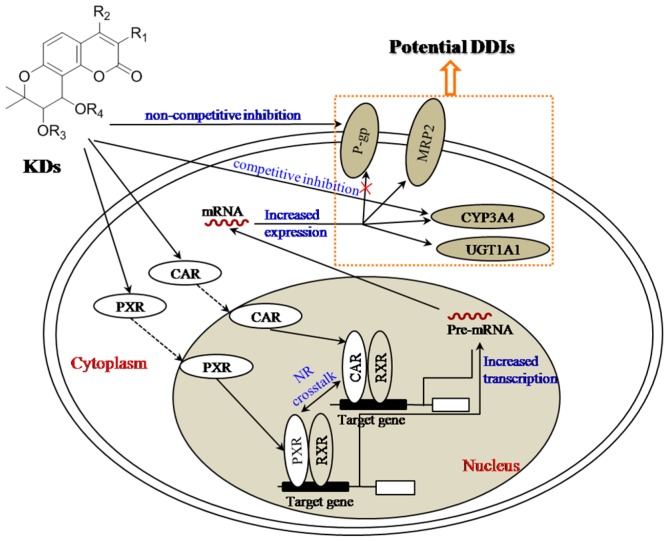
Schematic illustration of activation mechanisms of the potential drug-drug interactions (DDIs) initiated by KDs via CAR- and PXR-mediated pathways.

Collectively, the KDs-initiated drug-drug interaction potency is summarized in Figure 8. P-gp, CYP3A4, UGT1A1, and MRP2 were regarded to offer pivotal contribution. PXR- and CAR-mediated pathways were responsible for the upregulation of CYP3A4, UGT1A1, and MRP2 by KDs, however, without affecting the expression level of P-gp, while non-competitive and competitive inhibition occurred for P-gp and CYP3A4, respectively.

## 5. Conclusions and Perspectives

The scope of this review mainly focuses on summarizing the available pharmacokinetic and metabolic information on KDs. The predominant metabolic pathways are revealed as stepwise hydrolysis, stepwise oxidation, acyl-migration, and glucuronidation, while CYP3A, carboxylesterases, and UGTs offer primary contributions to these metabolic reactions. The knowledge concerning the SMRs suggests that the acyloxy groups at C-3′ and C-4′ play the determinant roles for the metabolic patterns of KDs, and preliminary information has indicated the introduction of hydrophilic substituents at C-3 and C-4 sites could lower the lipophicity, and thus strengthen the metabolic stability. A series of evaluations have demonstrated that KDs could not only regulate the expression of both metabolizing enzymes and transporters, but also affect the activities of P-gp and CYP3A via a competitive or non-competitive manner, indicating that attention should be paid to the potential for DDIs during clinical trials of KDs. Above all, in the future innovative efforts should be made to counterbalance the dramatic pharmacological activities and the metabolic features of KDs, as well as the potential DDIs, during structural optimization. 

## Figures and Tables

**Table 1:** Pharmacokinetic parameters of khellactone derivatives *in vivo*.

Comp.	Route	CL (mL/min/kg)	V_d_ (L/kg)	AUC (μg·min/L)	*t*_1/2_ (min)	*C*_max_ (ng/mL)	*t*_max_ (h)	MRT_0–t_ (h)	F (%)	Ref.
Ref. 13-4 ^a^	i.v.	0.01 ± 0.003	2.34 ± 0.31	195,270.08	97 ± 11	N.A.	N.A.	N.A.	N.A.	[17]
Ref. 13-4	i.g.	0.058 ± 0.01	8.19 ± 2.60	348,067.29	167 ± 30	N.A.	N.A.	N.A.	17.8	[17]
CMDCK	i.v.	0.044 ± 0.010	8.14 ± 5.88	47,082 ± 10,464	120 ± 60	N.A.	N.A.	N.A.	N.A.	[47]
CMDCK	i.g.	N.A.	N.A.	29,874 ± 4524	200.4 ± 31.8	105.7 ± 73.3	0.4 ± 0.1	N.A.	15.8 ± 2.1	[47]
HMDCK ^b^	i.v.	0.050 ± 0.0017	0.008 ± 0.002	558.4 ± 8.71	N.A.	N.A.	N.A.	1.235 ± 0.618	N.A.	[48]
HMDCK ^b^	i.g.	0.33 ± 0.083	0.079 ± 0.039	57,978 ± 13,182	N.A.	530.7 ± 120.8	0.58	1.983 ± 0.478	17.3	[48]
Ref. 48-5	i.g.	0.55 ± 0.10	0.252 ± 0.130	31,302 ± 8016	N.A.	284.1 ± 74.73	0.50	3.092 ± 0.454	10.3	[48]
Ref. 48-6	i.g.	1.7 ± 0.33	0.669 ± 0.422	9474 ± 2576.4	N.A.	80.9 ± 18.80	0.38	2.658 ± 0.681	3.3	[48]
Ref. 48-7	i.g.	0.77 ± 0.22	0.373 ± 0.193	21,252 ± 6336	N.A.	254.6 ± 32.45	0.25	3.769 ± 0.063	7.2	[48]
Ref. 48-8	i.g.	0.57 ± 0.067	0.102 ± 0.011	35,334 ± 4323.6	N.A.	398.5 ± 29.00	0.50	1.517 ± 0.207	12.4	[48]
Ref. 48-9	i.g.	0.62 ± 0.017	0.467 ± 0.063	23,046 ± 372.6	N.A.	163.6 ± 13.56	0.25	3.581 ± 0.307	7.5	[48]
Ref. 48-10	i.g.	0.27 ± 0.12	0.045 ± 0.017	77,790 ± 26,256	N.A.	621.5 ± 79.67	0.75	1.793 ± 0.181	25.7	[48]
Ref. 48-12	i.g.	0.43 ± 0.083	0.049 ± 0.020	46,536 ± 9774	N.A.	529.0 ± 192.9	0.50	1.286 ± 0.085	15.8	[48]
Ref. 48-13	i.g.	0.67 ± 0.17	0.123 ± 0.058	28,668 ± 7722	N.A.	191.1 ± 2.48	0.75	2.136 ± 0.123	9.8	[48]
PA	i.v.	N.A.	N.A.	37,835.6 ± 5871.6	51.18 ± 9.02	N.A.	N.A.	N.A.	N.A.	[37,39,49]
Pteryxin	i.g.	N.A.	N.A.	4128.08	87.78	976.04	2.00	6.732	N.A.	[38,41]
*d*PB	i.v.	9.6 ± 3.2	N.A.	1,088,700 ± 375,900	7.14 ± 2.14	N.A.	N.A.	N.A.	N.A.	[36]
*d*PA	i.v.	N.A.	N.A.	80,346 ± 8724	109.2 ± 51.6	N.A.	N.A.	1.61 ± 0.58	N.A.	[40]
*l*PA ^c^	i.v.	N.A.	N.A.	N.A.	N.A.	N.A.	N.A.	N.A.	N.A.	[40]
*l*CK ^d^	i.g.	N.A.	N.A.	191,172 ± 89,460	352.2 ± 169.8	345.6 ± 204.0	0.71 ± 0.19	10.0 ± 3.35	N.A.	[40]
*l*CK ^e^	i.g.	N.A.	N.A.	512,820 ± 235,470	353.4 ± 157.2	1156.3 ± 637.6	0.72 ± 0.53	10.3 ± 3.95	N.A.	[40]
*d*CK ^f^	i.g.	N.A.	N.A.	46,536 ± 22,890	409.2 ± 180.6	108.5 ± 38.2	0.43 ± 0.35	11.0 ± 4.46	N.A.	[40]
*l*CK ^f^	i.g.	N.A.	N.A.	177,612 ± 98,910	427.8 ± 266.4	685.1 ± 254.3	0.36 ± 0.20	13.7 ± 5.84	N.A.	[40]
*d*TK ^g^	i.g.	N.A.	N.A.	29,280 ± 6462	526.2 ± 69	55.5 ± 31.3	4.83 ± 1.83	N.A.	N.A.	[8]
*l*CK ^g^	i.g.	N.A.	N.A.	213,414 ± 54,096	420 ± 85.2	468 ± 233	1.08 ± 1.14	N.A.	N.A.	[8]
*d*CK ^g^	i.g.	N.A.	N.A.	50,982 ± 10,110	420.6 ± 84	362 ± 224	1.08 ± 1.14	N.A.	N.A.	[8]
*d*PA ^g^	i.g.	N.A.	N.A.	3468 ± 1620	1666.2 ± 2049	19.8 ± 11.3	0.38 ± 0.56	N.A.	N.A.	[8]
*d*PB ^g^	i.g.	N.A.	N.A.	7932 ± 2088	526.2 ± 69	10.3 ± 5.42	4.83 ± 1.83	N.A.	N.A.	[8]
*d*PE ^g^	i.g.	N.A.	N.A.	7734 ± 954	526.2 ± 69	5.35 ± 0.41	4.83 ± 1.83	N.A.	N.A.	[8]

^a^: follow the number named in corresponding references; ^b^: (3′,4′)-3-hydroxymethyl-4-methyl-3′,4′-di-(*S*)-camphanoyl-(+)-*cis*-khellactone; ^c^: parent compound could only be detected before time 10 min; ^d^: the parameters were obtained after oral treatment of PA; ^e^: the parameters were obtained after oral treatment of *d*PA; ^f^: the parameters were obtained after oral treatment of *l*PA; ^g^: the parameters were obtained after oral treatment of Peucedani Radix extract; N.A.: not archived in corresponding references.

**Table 2:** The bi-directional *P*_app_ values of some khellactone derivatives in the Caco-2 cell monolayer model.

Compound	*P*_app AP__→__BL_ (×10^−6^, cm/s)	*P*_app BL__→__AP_ (×10^−6^, cm/s)	*P*_app BL__→__AP_/*P*_app AP__→__BL_	Ref.
*d*PB	1.25 ± 0.05	1.33 ± 0.13	1.06	[50]
*l*PB	15.27 ± 0.45	13.82 ± 1.37	0.90	[51]
*d*PA	22.5–30.3	16.5–19.7	0.6–0.8	[46]
*l*PA	20.1–28.2	15.8–18.8	0.6–0.8	[46]
CMDCK	23.2 ± 1.76	12.1 ± 1.37	0.52	[17,47]

**Table 3:** Kinetic parameters of khellactone derivatives *in vitro*.

Comp.	*t*_1/2_ (min)	CL_int_ (mL/min/mg)	CL_h_ (mL/min/kg)	Q_h_ (mL/min/kg)	K_m_ (μmol/L)	V_max_ (pmol/min/mg)	Ref.
Ref. 38-1 ^a^	2.4	2.9	-	-	-	-	[59]
Ref. 38-2	5.1	1.4	-	-	-	-	[59]
Ref. 38-3	1.5	4.6	-	-	-	-	[59]
Ref. 38-4	2.1	3.3	-	-	-	-	[59]
Ref. 38-5	3.7	1.9	-	-	-	-	[59]
Ref. 38-6	2.0	3.5	-	-	-	-	[59]
Ref. 38-7	3.4	2.0	-	-	-	-	[59]
Ref. 38-8	5.2	1.3	-	-	-	-	[59]
Ref. 38-9	2.2	3.2	-	-	-	-	[59]
Ref. 38-10	2.0	3.5	-	-	-	-	[59]
Ref. 38-11	2.1	3.3	-	-	-	-	[59]
Ref. 38-12	^b^	^b^	-	-	-	-	[59]
Ref. 38-13	5.7	1.2	-	-	-	-	[59]
Ref. 38-14	4.3	1.6	-	-	-	-	[59]
Ref. 48-3	17.92	0.39	-	-	-	-	[48]
Ref. 48-5	34.61	0.20	-	-	-	-	[48]
Ref. 48-6	35.27	0.20	-	-	-	-	[48]
Ref. 48-7	29.07	0.24	-	-	-	-	[48]
Ref. 48-8	25.93	0.27	-	-	-	-	[48]
Ref. 48-9	3.90	1.78	-	-	-	-	[48]
Ref. 48-10	49.26	0.14	-	-	-	-	[48]
Ref.48-11	30.09	0.23	-	-	-	-	[48]
Ref. 48-12	30.77	0.23	-	-	-	-	[48]
Ref. 48-13	26.54	0.26	-	-	-	-	[48]
CMDCK (HIM)	25.7	0.012	3.3	-	45.6	0.33	[56]
CMDCK (HLM)	5.62 ± 0.57	0.31 ± 0.031	19.4 ± 0.12	20.7	14.3	1.78	[58]
CMDCK (CYP3A4)	6.84 ± 1.55	-	-	-	12.1	1.58	[58]
PA (HLM)	30.13 ^c^	0.27	0.12	-	-	-	[54]
*l*CK		1.29			0.02 ± 0.004	25.8 ± 2.70	[60]
CAK-4					4.33 ± 1.40	0.402 ± 0.0715	[40]
CAK-3					9.97 ± 3.55	0.663 ± 0.165	[40]
PA(RLM)	8.19 ^c^	0.24 ± 0.02	-	-	64.1 ± 4.22	0.26 ± 0.036	[37,54,57]
*d*PA(HLM)	22.65 ^c^	0.20	-	-	17.83 ± 15.02	-	[9,60]
*d*PA(RLM)	10.24 ^c^	-	-	-	-	-	[9]
*l*PA(HLM)	31.09 ^c^	0.28	-	-	-	-	[9]
*l*PA(RLM)	3.01 ^c^	-	-	-	-	-	[9]

^a^: follow the number named in corresponding references; ^b^: parent compound could not be detected at time 0 min; ^c^: calculated using the CL_int_ value documented in corresponding references; -: not archived in corresponding references.
